# Prevalence and Predictors of Medical Mistrust Among Socioeconomically and Racially Diverse Cancer Patients in Philadelphia

**DOI:** 10.3390/cancers17040649

**Published:** 2025-02-14

**Authors:** Michael J. Hall, Cindy Y. Park, Karen J. Ruth, Patrick J. A. Kelly, Katie Singley, Caseem C. Luck, Yana Chertock, Sarah Bauerle Bass

**Affiliations:** 1 Department of Clinical Genetics, Fox Chase Cancer Center, Philadelphia, PA 19111, USA; 2 Lewis Katz School of Medicine, Temple University Health System, Philadelphia, PA 19140, USA; 3 Biostatistics and Bioinformatics, Fox Chase Cancer Center, Philadelphia, PA 19111, USA; 4 College of Public Health, Temple University, Philadelphia, PA 19122, USAkatie.singley@temple.edu (K.S.);

**Keywords:** medical mistrust, cancer burden, underserved populations, safety net institutions, race/ethnicity, cancer health disparities, health literacy, provider–patient communication

## Abstract

**Background/Objectives**: Medical mistrust (MM) is associated with adverse health outcomes, but few studies have assessed MM in cancer patients. MM is frequently measured using the Medical Mistrust Inventory (MMI), measuring institutional MM (e.g., government), or the Group-Based Medical Mistrust Scale (GBMMS), measuring race-based MM. We sought to assess the prevalence of MM among cancer patients diverse by age, sex, race/ethnicity, and socioeconomic status (SES), recruited from an urban safety net hospital and a suburban comprehensive cancer center. **Methods**: Patients completed a one-time survey. The primary outcome was MM as measured by the GBMMS and MMI tools. Covariates included demographics, treatment campus (urban vs. suburban), and psychosocial measures relevant to MM. **Results**: Purposeful sampling recruitment resulted in 200 participants (survey completion: 74.6%). The median age was 60 years, with 62% female, 45% African-American, 15% Hispanic, 47.5% education ≤ HS diploma, and 51.5% income ≤ USD 50,000/yr. Elevated MMI and GBMMS scores (moderate-to-high) were seen, respectively, in Hispanic (20.7% and 33.4%) and African-American (AA) patients (31.8% and 48.9%), compared with White patients (14.3% and 9.9%). The MMI and GBMMS tools captured complimentary aspects of MM in cancer patients (Spearman’s 0.531, *p* < 0.0001). MMI was associated with lower education (0.034) and race (*p* = 0.04), while GBMMS was strongly associated with race (*p* < 0.001), urban campus (*p* = 0.035), and mistrust of government/health organization information (both *p* < 0.05). Higher MMI/GBMMS scores were both associated with research mistrust and mistrust of information from physicians. **Conclusions**: Institutional and race-based MM are prevalent among cancer patients diverse by age, sex, race/ethnicity, and SES. Lower education was associated with institutional MM but not race-based MM.

## Simple Summary

Patients with medical mistrust are less likely to seek critical healthcare and have been shown to have poorer health outcomes. Medical mistrust is often measured by a scale focused on government/institution mistrust or a different scale focused more on race-based mistrust. We conducted a survey to examine medical mistrust in a group of 200 cancer patients, diverse by age, race/ethnicity, sex, and diagnosis, receiving care at urban and suburban sites of the same cancer center in Philadelphia, and we measured medical mistrust with two scales. Institutional and race-based mistrust were prevalent and captured different facets of mistrust. Medical mistrust was found in all racial/ethnic groups but was more frequently elevated in African-American and Hispanic patients than White patients. Institutional but not race-based mistrust was associated with lower education. Both institutional and race-based mistrust were associated with mistrust of research and mistrust of information from physicians (compared with other information sources).

## 1. Introduction

Medical mistrust is defined as the “belief that the entity that is the object of mistrust is acting against one’s best interest or well-being” [1]. It is more than simply an absence of trust; rather, it connotes a frame of view in which skepticism towards healthcare equity, including access, quality, and even provider intentions, is questioned [2]. Medical mistrust has been studied in a variety of populations and sociomedical contexts, but in cancer care has largely been focused on its prevalence among African-American (AA) patients, most notably women with breast cancer and men with prostate cancer [3,4,5,6,7,8]. Several studies of medical mistrust among AA patients considering cancer screening or germline genetic testing have also been conducted, with a smaller number of studies examining Hispanic and Native American populations, rural Americans, and sexual and gender minorities [9,10,11,12,13,14,15,16,17,18].

The prevalence of medical mistrust is well documented among AAs and has been associated with a variety of adverse health outcomes [19,20]. Among AAs, medical mistrust is frequently attributed to the extensive history of race-based discrimination at a national level and systemic currents of social discrimination and inequalities over time [21]. More recently, medical mistrust among AAs has been exacerbated by medical misinformation and divisive messages spread through social media, word of mouth, and other means [1,19,20]. A growing literature has also documented medical mistrust in other marginalized groups, including sexual gender minorities and individuals living with HIV infection. Conspiratorial messaging and thinking related to healthcare equity have been highlighted in these populations, demonstrating medical mistrust as a barrier to improving health outcomes and patient–physician relationships in these already marginalized groups [1,2,22].

Two commonly cited measures of medical mistrust are scales developed by LaVeist [23] (Medical Mistrust Inventory or MMI) and Thompson [24] (Group-Based Medical Mistrust Scale or GBMMS). The former is a 17-item scale with statements elucidating medical mistrust related to perceptions of government and healthcare institutions. The latter is a 12-item scale that focuses on medical mistrust rooted in perceptions of race/ethnicity-based discrimination within healthcare, and it includes three subscales (*suspicion*, *discrimination*, and *lack of support*). Higher scores on the MMI and GBMMS scales have been associated with negative health outcomes, such as lower uptake of cancer screening and poorer quality of life [6,20,23].

A paucity of literature has explored medical mistrust in the cancer population. Considered in the light of the COVID-19 pandemic and the resulting heightened social skepticism toward government-endorsed lockdowns and vaccination policies, medical mistrust among patients from diverse racial/ethnic backgrounds undergoing current cancer treatment would not be surprising, especially with the pandemic reducing access to medical care [25]. Similarly, current race-based social movements associated with highly publicized police brutality (e.g., Black Lives Matter) and subsequent judicial inequalities support that social mistrust is an important construct that has roots in race-based concerns of discrimination and anti-institutional sentiment (government, law enforcement) [26]. Nonetheless, the comparative prevalence of government/healthcare institution and race-based medical mistrust has not been examined in a demographically diverse sample of cancer patients.

Cancer patients face a growing number of complex medical decisions where shared decision-making with providers is critical [27]. Management decisions informed by genetic genetics/genomics, areas that may be particularly sensitive for some racial/ethnic minority patients [28], are commonplace in modern cancer care [29]. Little is known about factors that may be associated with medical mistrust among cancer patients and that may serve as barriers to provider–patient communication, shared decision-making, and optimal health outcomes.

The objective of the current study was to collect data on the prevalence of institutional and race-based medical mistrust in a demographically diverse population of cancer patients and to understand the overlap and divergences of institutional and race-based medical mistrust and their relationship to demographic factors and psychosocial measures relevant to mistrust in healthcare. We hypothesized that both institutional mistrust (MMI) and race-based mistrust (GBMMS) are prevalent among cancer patients and would strongly associate with demographic factors, conspiratorial beliefs, health information mistrust, and negative attitudes about research. Moreover, by identifying predictors of medical mistrust in the cancer population, our long-term goal is to develop and test novel interventions toward medical mistrust, which may be beneficial for mitigating associated poor health outcomes.

## 2. Materials and Methods

We conducted a cross-sectional survey of patients from a comprehensive cancer center and a safety net hospital in Philadelphia, PA (June 2022–December 2022). Approximately half of recruited participants were receiving care at a university-based safety net hospital in an economically depressed area (i.e., urban site), with many of the patients identifying as racial/ethnic minorities and being uninsured or Medicaid recipients. The remaining participants were recruited from a suburban comprehensive cancer center in the same health system that serves a mixed urban/suburban population with substantial racial/ethnic and socioeconomic diversity from poorer urban neighborhoods and wealthier suburban communities (i.e., suburban site). Participants were recruited in outpatient clinics using a purposeful sampling approach to assure ample variability by age, sex, and race/ethnicity. To guide recruitment, a sampling grid of target characteristics and sample sizes was developed (Qualtrics), and individuals meeting the criteria were enrolled and target buckets were filled until the minimum number for each category was achieved. Overall, of the 272 eligible patients approached who agreed to review the consent, four refused to participate (consent rate 98.5%), and among those who provided informed consent, 74.6% (200/268) completed the survey, fulfilling all target goals. Our sample size was informed from prior literature [30] and was calculated to have the power to detect moderate effect sizes for predictive factors of medical mistrust.

### 2.1. Outcome Measures

The one-time survey (127 items) took approximately 25–30 min to complete, and participants received a USD 20 gift card. The survey included the following outcome measures and covariates selected as relevant to medical mistrust.

#### 2.1.1. Medical Mistrust

The primary outcome was medical mistrust (MM) quantified by the LaVeist and Thompson scales. We modified the original 4-point Likert scale used by LaVeist [23] (strongly disagree to strongly agree) to an 11-point scale (0 to 10, strongly disagree to strongly agree), in line with our prior research assessing mistrust among AA cancer patients [14]. The GBMMS scale measures race/ethnicity specific MM on a 5-point Likert scale (strongly disagree to strongly agree) and has three subscales (*suspicion*, *discrimination*, and *lack of support*).

#### 2.1.2. Patient Characteristics

Age, sex, race/ethnicity, marital status, education, income, and insurance were queried, as was cancer stage awareness. Race/Ethnicity was categorized as Black or AA non-Hispanic, White non-Hispanic, any Hispanic, and other.

#### 2.1.3. Health Literacy

Health literacy was measured using a single-item literacy screener (SILS) developed by Chew et al. [31]: “How often do you need to have someone help you when you read instructions, pamphlets, or other written material from your doctor or pharmacy” (1—never, 2—rarely, 3—sometimes, 4—often, and 5—always). Scores >2 were considered positive. SILS has been shown to be an effective health literacy screen in chronically ill low health literacy populations [32].

#### 2.1.4. Conspiratorial Thinking

Conspiratorial thinking was assessed using a single-item measure developed by Lantian et al. [33]: “I think the official version of events given by authorities very often hides the truth.” Response was disagreement/agreement on an 11-point Likert scale.

#### 2.1.5. Trust in Information Sources

Trust in information sources was assessed with items developed from the Health Information National Trends Survey (HINTS) [34]. This measure includes 13 items assessing trust (1—not at all to 4—a lot) in information sources for cancer from sources including healthcare professionals, family/friends, cancer patients, media sources, the government, religious/charitable organizations, or patient testimonials.

#### 2.1.6. Trust in Medical Research

Trust in medical research items (n = 10) were adapted from our past research, including statements related to physician researchers: “Medical research is just being done to make money”, “I think medical researchers use patients as guinea pigs”, and “Racial/ethnic minorities are discriminated against in medical research” [14].

The study was approved by the Fox Chase Cancer Center Institutional Review Board (IRB 22-8003).

### 2.2. Statistical Analysis

Characteristics were compared by treatment site using chi-square or Fisher’s exact tests for categorical variables and by *t*-tests or Wilcoxon rank-sum tests for continuous variables. The correlation of MMI and GBMMS scores was measured using Spearman’s coefficient. Within characteristic categories, the median and mean medical mistrust scores are shown. ANOVA general linear models were used to compare mistrust scores within characteristic categories and to adjust for demographic and SES covariates in multivariable models; residuals from these models were examined to assess the normality assumption. MMI and GBMMS scores were also categorized as trust, unsure, and high mistrust based on the score relative to the corresponding Likert scale. For demographic and SES characteristics with significant associations with mistrust scores, we used multivariable analyses to assess the partial associations, using *p* < 0.1 for inclusion in the multivariable model. For MMI, both education and income met these criteria, but because of their high collinearity, income was not included. For conspiratorial thinking and trust in medical research, adjusted mean scores within each MMI and GBMMS category were compared with ANOVA tests, controlling for significant demographic and SES factors. For other psychosocial measures, MMI and GBMMS scores were compared within the psychosocial measure using multivariable ANOVA models, adjusting for covariates as above. For the 13 items in the trust in information sources survey, *p*-values are only reported for items where both MMI and GBMMS were not statistically significant. Mistrust scores were not calculated for surveys with 2 or more missing items for GBMMS and 4 or more missing items for MMI. Analyses were performed using SAS software (9.4) and R (version 4.4.0), with two-sided tests and statistical significance being set at *p* < 0.05. Dot plot figures (Figure 2a,b and Figure S1) were created using R (version 4.4.0). For Figure 2a,b, race = other was omitted due to the small sample (n = 8).

## 3. Results

### 3.1. Participant Characteristics

The participants (n = 200) included 95 patients from the urban campus and 105 patients from the suburban campus. Demographic characteristics can be seen in Table 1. Purposeful sampling was successful in recruiting participants diverse by age, sex, and race/ethnicity.

Mean participant ages were 60.7 years and 59.3 years at the urban and suburban campuses, respectively (*p* = 0.43), with similar distributions. More participants from the urban campus were Black/non-Hispanic (58.9% vs. 33.3%) and Hispanic (23.2% vs. 7.6%) (*p* < 0.001). Participants from both campuses were more often female; however, participants that were urban campus recruits were more often single (*p* < 0.001). Socioeconomic status (SES) varied by campus: 36.8% of participants from the urban campus reported household income < USD 10,000, and more than two-thirds reported educational attainment ≤ high-school diploma/GED. Significantly more participants at the suburban campus reported annual income > USD 50,000 and had a 4-year college degree or higher (both *p* < 0.001). Notably, 42.1% of participants from the urban campus and 22.9% of participants from the suburban campus were uncertain of their cancer stage (*p* = 0.006).

### 3.2. Primary Outcomes

#### 3.2.1. Prevalence of Medical Mistrust

**Figure 1:**
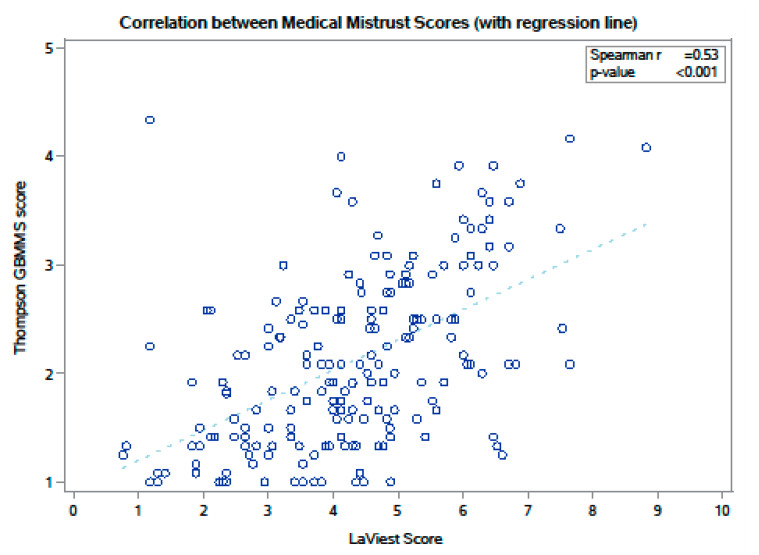
Scatterplot matrix of MMI and GBMMS scores.

The mean MMI score was 4.28 (0–10 scale; SD 1.54; median 4.29) and mean GBMMS score was 2.09 (1–5 scale; SD 0.79; median 2.04). GBMMS subscale scores were as follows: *Suspicion:* mean, 1.51 (SD 0.75); median 1.33; *Discrimination:* mean 2.57 (SD 1.14); median 2.67); and *Lack of support*: mean 2.17 (SD 0.92); median 2.33. Comparing these measures, the Spearman correlation coefficient was 0.531 (*p* < 0.0001), suggesting the two measures captured shared and unique elements of medical mistrust in the sample. The Spearman correlation coefficients for MMI and the GBMMS subscales were: *Suspicion* (0.47), *Discrimination* (0.36), and *Lack of Support* (0.50) (all *p* < 0.001). The Figure 1 scatterplot matrix visually demonstrates to what degree the mistrust measures overlap and differ.

#### 3.2.2. Demographic Predictors of Medical Mistrust

The MMI and GBMMS scales demonstrated significant associations with demographic and disease-related characteristics (Table 2). Overall, 14.3% of White, 20.7% of Hispanic, and 31.8% of AA participants had an unsure or high MMI score, while moderate and higher scores by GBMMS were found in 8.5% and 1.4% of White participants, 37.8% and 11.1% of AA participants, and 26.7% and 6.7% of Hispanic participants, respectively.

**Figure 2:**
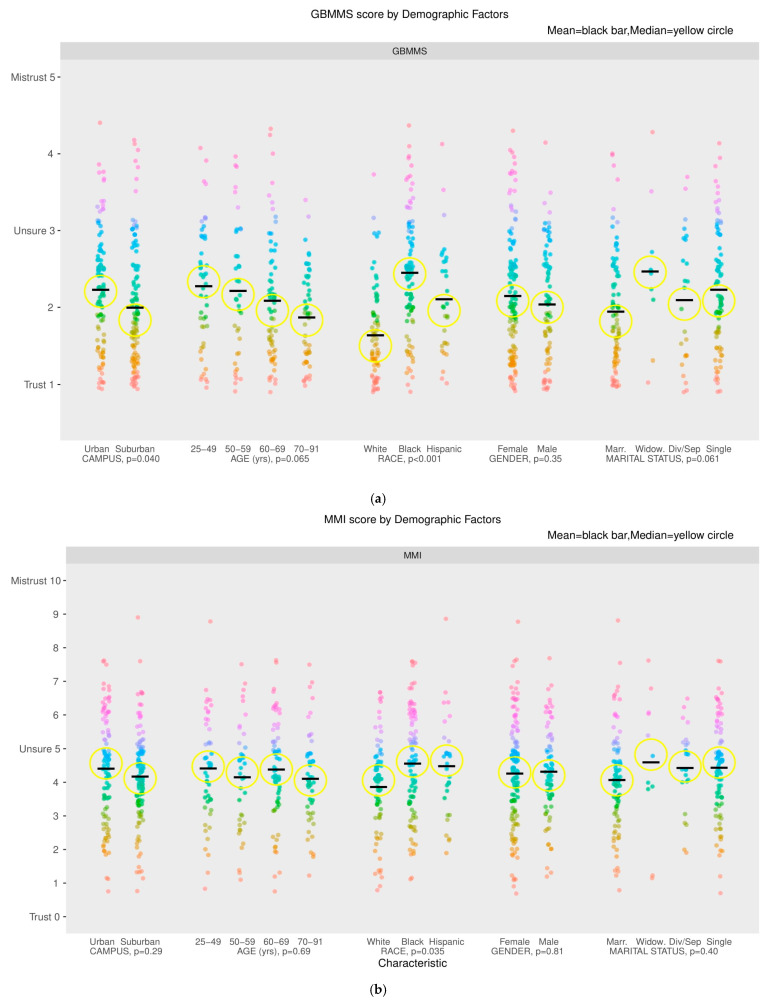
(**a**) Association of treatment site and demographic variables to GBMMS score; (**b**) association of treatment site and demographic variables to MMI score.

MMI was associated with race (*p* = 0.04) and educational attainment (0.034), while GBMMS was associated with race (*p* < 0.001) and treatment location, with higher GBMMS scores occurring among patients treated at the urban campus compared with the suburban location (*p* = 0.035). Both scales demonstrated that patient race/ethnicity, specifically AA or Hispanic, was associated with higher medical mistrust (GBMMS *p* < 0.001, MMI *p* = 0.04). Younger patients and lower income individuals also generally had higher MMI and GBMMS scores. MMI score was higher among those who were “unsure” of their cancer stage (*p* = 0.041), while GBMMS was higher in those who reported they were “early stage” and in those who were “unsure” of their cancer stage (*p* = 0.02). Neither measure was associated with cancer histology. Associations of demographic figures with outcomes can be appreciated visually in Figure 2 (GBMMS, MMI) below.

#### 3.2.3. Multivariable Models

Multivariable analyses were conducted to assess whether the association of the mistrust scores with significant (*p* < 0.1) demographic and SES factors remained significant after controlling for the other covariates. The LaVeist MMI score model included race, education, and cancer stage, while the Thompson GBMMS score model included campus, age, race, marital status, and cancer stage. In the adjusted MVA model examining MMI, race/ethnicity (*p* = 0.096), and cancer stage (*p* = 0.12), both lost significance while educational attainment remained significant (*p* = 0.032). In the MVA model examining GBMMS with adjustment for other covariates, race (*p* < 0.001) remained significant.

### 3.3. Associations with Measures Relevant to Medical Mistrust

#### 3.3.1. Psychosocial Measures Related to Medical Mistrust

We identified associations among several psychosocial measures and medical mistrust in univariate analyses. *Conspiratorial thinking* was prevalent in our cohort. The mean score was 6.53 (SD 3.25, median 6, range 0–10), and was strongly associated with MMI (*p* = 0.0005) and GBMMS (*p* = 0.0006). *Trust of information sources* was also associated with mistrust; among the information sources queried by HINTS, *trust of healthcare professionals* was inversely associated with MMI (*p* = 0.0037) and GBMMS (*p* < 0.0001). However, *trust of healthcare organizations* was inversely associated only with MMI (*p* = 0.0023). *Trust in medical research* was also strongly inversely associated with MMI and GBMMS (both *p* < 0.001). In multivariate analyses (Table 3 and Table 4), the majority of the associations remained significant, including trust in information from a doctor/health professional (*p* = 0.002 for MMI, *p* < 0.001 for GBMMS) and the association of trust in medical research with MMI and GBMMS (both *p* < 0.001).

#### 3.3.2. Associations with GBMMS Subscales Suspicion, Discrimination, and Lack of Support

GBMMS subscale scores showed marked variability in their associations with demographic, disease-related, and psychosocial predictors (Supplementary Tables S1 and S2). Variability in the GBMMS subscales by MMI score, campus, race/ethnicity, and stage awareness are visualized in Figure S1.

## 4. Discussion

Medical mistrust is associated with adverse health outcomes across racial/ethnic minorities, rural populations, and sexual gender minorities [1,23]. Much of the research on medical mistrust has examined AA and other minority populations, often through the lens of race/ethnicity-based mistrust and often employing the Thompson GBMMS measure [24]. Nonetheless, our results demonstrate that medical mistrust, while higher among minority cancer patients, is not limited to this group. In our sample of cancer patients of diverse age, sex, race/ethnicity, and SES treated in urban and suburban Philadelphia, institutional medical mistrust (measured by MMI [23]) was notably elevated among 14.3% of White, 20.7% of Hispanic, and 31.8% of AA participants. Race-based medical mistrust (measured by GBMMS) was more prevalent than institutional mistrust, with moderate and high scores in 8.5% and 1.4% of White participants, 37.8% and 11.1% of AA participants, and 26.7% and 6.7% of Hispanic participants, respectively.

Our findings suggest that medical mistrust in cancer patients is multi-faceted. Institutional medical mistrust exists regardless of race/ethnicity; however, concerns about systemic and institutional health inequities linked to race/ethnicity may further magnify mistrust for minority patients. Indeed, on the scatterplot matrix comparing MMI and GBMMS (Figure 1), it is the *lack of support* subscale of GBMMS that correlates most strongly with MMI, supporting the notion that institutional medical mistrust in part may correlate with disenfranchisement and marginalization [35,36,37].

Apart from the small but statistically significant differences in MMI and GBMMS scores across race/ethnicity, only lower educational attainment was associated with higher MMI/institutional mistrust, with significance being maintained after multivariable adjustment (*p* = 0.032). This reflects previous research identifying lower educational attainment as a predictor of medical mistrust [19,38] but differs from other studies [19,38] where lower household income and federal insurance were also associated with higher mistrust. Unsurprisingly, our urban campus participants, who were > 80% AA or Hispanic, had higher race-based medical mistrust by GBMMS.

An unexpected finding was the significant prevalence of stage uncertainty—nearly one-third of participants (42.1% urban, 22.9% suburban) reported uncertainty of their cancer stage, with uncertainty associated with both MMI (*p* = 0.045) and GBMMS (*p* = 0.025) in univariate but not multivariable analyses (*p* = 0.12 and *p* = 0.16, respectively). We previously identified high rates of stage uncertainty in a sample of AA cancer patients and also identified an association of stage uncertainty with higher mistrust [14]. Our findings suggest that stage uncertainty may be particularly common among AA cancer patients, with an indirect relationship with medical mistrust. While stage uncertainty might at face value appear to reflect low health literacy, health literacy was not associated with either MMI or GBMMS in the current study. Alternatively, stage uncertainty may signify a poorer communication with and relationship with the oncology team, who would usually provide this information, due to mistrust of healthcare providers as an information source [39]. Consistent with this, we found that both MMI and GBMMS results were significantly associated with low trust of information from healthcare providers in univariate and adjusted analyses.

Controlling for other factors, lower trust in information from government and health agencies was associated with MMI and GBMMS (Table 4), as was mistrust towards medical research (Table 3). While mistrust of medical research might seem to be synonymous with institutional mistrust, the undeniable history of medical experimentation in underserved/minority populations in the US [36,40] lends credence to a facet of race/ethnicity-based mistrust towards research [1,40]. However, Jaiswal contends that medical mistrust is more nuanced than only a historically rooted explanation [1]. For example, social media has been shown to negatively contribute to COVID-19 vaccine hesitancy in AAs and treatment adherence for patients with HIV [1,19,20,41]. One explanation may be that patients obtain poor information, i.e., misinformation and conspiratorial beliefs, through social media, which then influences their behaviors. Social media has been strongly implicated in fueling conspiratorial thinking [42], and our study found strong associations of institutional and race-based mistrust to conspiratorial thinking. Similarly, mounting political polarization coupled with public health crises may further fuel medical mistrust toward institutions like government and healthcare institutions [43,44,45]. These are areas for further exploration in research [43,44,45].

Our study recruited a small number of Spanish-speaking and first-generation Hispanic patients, limiting power in assessing mistrust in these populations [46,47]. In addition, because we recruited in Philadelphia only, our findings should be replicated in other settings where cancer patients receive care. Finally, as a cross-sectional survey, we are only able to measure associations with medical mistrust. Further research investigating causality and methods to address institutional and race-based medical mistrust are critically needed in response to the negative impact of misinformation on provider–patient relationships and in the setting of the complex healthcare decisions necessary in cancer care.

## 5. Conclusions

Institutional and race-based medical mistrust exist in a substantial minority of cancer patients and define both overlapping and independent facets of mistrust in patients diverse by age, sex, race/ethnicity, and SES undergoing cancer treatment.

## Figures and Tables

**Table 1:** Characteristics of the study population (n = 200).

Characteristic	All		Urban Cancer Center		Suburban Cancer Center		
	N	%	N	%	N	%	*p*
**All**	200		95		105		
**Age (years)**							
Mean (SD)	60.0	(12.7)	60.7	(12.9)	59.3	(12.5)	0.43 *t*-test
Median (IQR)	62.0	(52–70)	63.0	(54–71)	61.5	(49–67)	0.35 Wilcoxon
**Age (years)**							0.48
25–49	48	24.0	21	22.1	27	25.7	
50–59	37	18.5	20	21.1	17	16.2	
60–69	64	32.0	27	28.4	37	35.2	
70–91	50	25.0	27	28.4	23	21.9	
Missing	1	0.5			1	1.0	
**Race/Ethnicity**							<0.001 FE
White non-Hispanic	71	35.5	13	13.7	58	55.2	
Black non-Hispanic	91	45.5	56	58.9	35	33.3	
Any Hispanic	30	15.0	22	23.2	8	7.6	
Other	8	4.0	4	4.2	4	3.8	
**Sex**							0.98
Female	124	62.0	59	62.1	65	61.9	
Male	76	38.0	36	37.9	40	38.1	
**Marital Status**							<0.001
Married/Partnered	83	41.5	23	24.2	60	57.1	
Widowed	11	5.5	7	7.4	4	3.8	
Divorced/Separated	24	12.0	11	11.6	13	12.4	
Single	81	40.5	53	55.8	28	26.7	
Missing	1	0.5	1	1.1			
**Education**							<0.001 FE
Grade school (<9th)	8	4.0	7	7.4	1	1.0	
Some high school	26	13.0	19	20.0	7	6.7	
HS diploma/GED	61	30.5	38	40.0	23	21.9	
Vocational	9	4.5	6	6.3	3	2.9	
Some college	41	20.5	14	14.7	27	25.7	
College graduate	31	15.5	7	7.4	24	22.9	
Graduate and higher	24	12.0	4	4.2	20	19.0	
**Income**							<0.001
<USD 10K	44	22.0	35	36.8	9	8.6	
USD 10K–USD 50K	59	29.5	27	28.4	32	30.5	
USD 50K–USD 100K	33	16.5	8	8.4	25	23.8	
USD 100K	24	12.0	1	1.1	23	21.9	
Missing/Decline	40	20.0	24	25.3	16	15.2	
**Health Insurance**							<0.001
Private only	51	25.5	10	10.5	41	39.0	
Medicare only	55	27.5	25	26.3	30	28.6	
Medicaid only	28	14.0	17	17.9	11	10.5	
Other only	23	11.5	22	23.2	1	1.0	
More than one kind	37	18.5	18	18.9	19	18.1	
Unsure/decline	6	3.0	3	3.2	3	2.9	
**Cancer stage**							0.006
Early stage	60	30.0	28	29.5	32	30.5	
Late stage	76	38.0	27	28.4	49	46.7	
Uncertain	64	32.0	40	42.1	24	22.9	

Abbreviations: SD, standard deviation; IQR, interquartile range; HS, high school; GED: graduate equivalency diploma; FE, Fishers Exact.

**Table 2:** Associations of treatment site, demographic, and disease characteristics with institutional mistrust (MMI) and race-based medical mistrust (GBMMS).

Measure	Median	MMI Mean (SD)	*p*	Median	GBMMS Mean (SD)	*p*
**Treatment site**			0.29			0.04
Urban campus	4.56	4.41 (1.66)		2.21	2.23 (0.79)	
Suburban campus	4.12	4.17 (1.43)		1.83	2.00 (0.80)	
**Demographic characteristics**						
Age (years)			0.69			0.065
25–49 (n = 48)	4.47	4.41 (1.60)		2.33	2.33 (0.81)	
50–59 (n = 37)	4.29	4.15 (1.65)		2.17	2.21 (0.89)	
60–69 (n = 64)	4.38	4.38 (1.56)		1.96	2.09 (0.83)	
70–91 (n = 49)	4.06	4.10 (1.39)		1.83	1.87 (0.63)	
Missing (n = 1)	5.18	5.18		2.83	2.83	
Race/Ethnicity			0.035			<0.001
Black or AA non-Hispanic (n = 90)	4.62	4.56 (1.60)		2.44	2.45 (0.79)	
White non-Hispanic (n = 71)	4.06	3.86 (1.46)		1.50	1.64 (0.59)	
Any Hispanic (n = 30)	4.65	4.48 (1.55)		1.96	2.11 (0.74)	
Other (n = 8)	4.21	4.20 (0.78)		2.75	2.40 (0.74)	
Gender			0.81			0.35
Female (n = 123)	4.29	4.26 (1.62)		2.08	2.15 (0.83)	
Male (n = 76)	4.21	4.32 (1.41)		2.00	2.04 (0.75)	
Marital status			0.40			0.061
Married	4.06	4.07 (1.56)		1.82	1.94 (0.75)	
Widowed	4.82	4.59 (2.10)		2.46	2.47 (0.95)	
Divorced/Separated	4.47	4.43 (1.26)		2.04	2.09 (0.84)	
Single	4.59	4.43 (1.51)		2.08	2.23 (0.80)	
Missing	2.65	2.65		2.17	2.17	
**Socioeconomic factors**						
Education			0.034			0.42
Less than 9th grade	3.09	3.51 (1.04)		1.70	1.79 (0.58)	
Some high school	4.76	4.70 (1.71)		2.08	2.15 (0.60)	
High school diploma/GED	4.58	4.60 (1.65)		2.17	2.22 (0.85)	
Vocational school	5.12	4.56 (1.98)		2.58	2.37 (0.83)	
Some college	3.88	3.69 (1.52)		2.08	2.13 (0.89)	
College degree	4.50	4.42 (1.19)		1.83	1.88 (0.69)	
Graduate degree and above	4.03	4.06 (1.19)		1.92	2.04 (0.88)	
Income			0.092			0.10
Less than USD 10,000	4.74	4.62 (1.66)		2.08	2.17 (0.76)	
USD 10,000–≤USD 25,000	4.44	4.28 (1.23)		2.29	2.15 (0.75)	
USD 25,000–≤USD 50,000	4.03	4.14 (1.62)		2.08	2.07 (0.65)	
USD 50,000–≤USD 75,000	3.94	3.86 (1.59)		1.58	1.86 (0.82)	
USD 75,000–≤USD 100,000	3.53	3.49 (1.42)		2.63	1.71 (0.65)	
USD 100,000 or greater	4.09	4.03 (1.40)		1.67	1.97 (0.89)	
Don’t know	5.59	5.20 (1.72)		2.48	2.59 (0.77)	
Decline	4.26	4.38 (1.55)		2.13	2.26 (0.93)	
Insurance			0.10			0.65
Private only	4.12	4.05 (1.63)		1.75	2.01 (0.88)	
Medicare only	4.00	4.02 (1.28)		2.08	2.05 (0.68)	
Medicaid only	4.65	4.55 (1.56)		2.08	2.21 (0.81)	
Other only	4.71	4.94 (1.55)		2.08	2.25 (0.80)	
More than one kind	4.18	4.20 (1.60)		1.75	2.02 (0.85)	
**Cancer stage**			0.044			0.028
Early (n = 60)	4.26	4.15 (1.64)		2.08	2.13 (0.85)	
Late (n = 75)	4.09	4.05 (1.37)		1.83	1.93 (0.70)	
Unsure (n = 64)	4.82	4.67 (1.59)		2.21	2.29 (0.83)	

Abbreviations: MMI, Medical Mistrust Inventory; GBMMS, Group-Based Medical Mistrust Scale; GED: graduate equivalency diploma.

**Table 3:** Associations of conspiratorial thinking and trust in medical research with institutional mistrust (MMI) and race-based medical mistrust (GBMMS), adjusted for demographic and SES factors.

	MMI Trust Category		GBMMS Trust Category	
Measure	Adjusted Mean (95%CI)	*p*	Adjusted Mean (95%CI)	*p*
**Measures of trust**				
***Conspiratorial thinking***		<0.001		0.006
Trust	6.6 (5.8–7.4)		7.0 (6.1–8.0)	
Unsure	6.9 (5.9–8.0)		8.5 (7.5–9.6)	
High mistrust	9.1 (8.0–10.3)		9.4 (7.5–11.3)	
***Trust in medical research***		<0.001		<0.001
Trust	3.0 (2.5–3.5)		3.3 (2.6–4.0)	
Unsure	4.9 (4.2–5.5)		5.1 (4.3–5.9)	
Mistrust	7.0 (6.3–7.8)		6.7 (5.3–8.1)	

Abbreviations: MMI, Medical Mistrust Inventory; GBMMS, Group-Based Medical Mistrust Scale. Models with GBMMS trust categories adjusted for site, age, race, marital status, and cancer stage. Models with MMI trust categories adjusted for race, education, and cancer stage.

**Table 4:** Association of health literacy and trust in information sources with institutional mistrust (MMI) and race-based medical mistrust (GBMMS), adjusted for demographic and SES factors.

	MMI Score		GBMMS Score	
	Adjusted Mean (95% CI)	*p*	Adjusted Mean (95%CI)	*p*
**Psychosocial measure**				
**Health literacy**		0.12		0.46
Never need help with medications	4.11 (3.7–4.52)		2.24 (2.03–2.45)	
Any help with medications	4.43 (3.98–4.88)		2.15 (1.91–2.39)	
**Trust in information sources**				
Doctor/Health professional		0.002		<0.001
Not at all/A little	5.05 (4.12–5.98)		2.68 (2.24–3.13)	
Some	4.93 (4.40–5.46)		2.66 (2.39–2.93)	
A lot	4.04 (3.69–4.38)		2.09 (1.91–2.27)	
**Family/friends**		0.018		0.57
Not at all/A little	4.60 (3.93–5.26)		2.32 (1.96–2.68)	
A little	4.52 (4.07–4.98)		2.23 (1.98–2.48)	
Some	3.89 (3.48–4.30)		2.12 (1.90–2.35)	
A lot	4.73 (4.12–5.34)		2.32 (2.00–2.64)	
**Radio**		0.067		0.026
Not at all/A little	4.54 (4.09–4.99)		2.33 (2.09–2.57)	
Some	4.19 (3.77–4.62)		2.15 (1.92–2.39)	
A lot	3.87 (3.35–4.39)		2.14 (1.87–2.4)	
**Government and health agencies**		0.049		0.015
Not at all	4.71 (3.99–5.43)		2.57 (2.21–2.93)	
A little	4.53 (3.96–5.09)		2.38 (2.10–2.66)	
Some	4.36 (3.93–4.79)		2.14 (1.92–2.37)	
A lot	3.83 (3.37–4.28)		2.06 (1.83–2.30)	
**Health organizations**		0.33		0.017
Not at all/A little	4.42 (3.83–5.00)		2.43 (0.70)	
Some	4.38 (3.94–4.82)		2.06 (0.77)	
A lot	4.06 (3.94–4.82)		2.02 (0.84)	
**Other sources**				
Other patients		0.10		0.11
Newspapers		0.11		0.64
Magazines		0.21		0.24
Internet		0.16		0.54
Television		0.33		0.12
Religious organizations		0.29		0.48
Charitable organizations		0.94		0.52
Patient testimonials		0.88		0.43

Abbreviations: MMI, Medical Mistrust Inventory; GBMMS, Group-Based Medical Mistrust Scale. GBMMS model categories adjusted for site, age, race, marital status, and cancer stage. MMI model categories adjusted for race, education, and cancer stage.

## Data Availability

Data are contained within the article or Supplementary Materials.

## Supplementary Materials

The following supporting information can be downloaded at: https://www.mdpi.com/article/10.3390/cancers17040649/s1, Figure S1. Distribution of GBMMS medical mistrust scores for LaVeist MMI, site of treatment, race and stage awareness stratified by suspicion, discrimination, and lack of support subscales. Table S1. Association of demographic factors with race-based mistrust (GBMMS) subscales of suspicion, discrimination, and lack of support. Table S2. Association of health literacy and trust in information sources with race-based discrimination (GBMMS) subscales suspicion, discrimination, and lack of support

## Abbreviations

The following abbreviations are used in this manuscript:

MM	Medical Mistrust
AA	African-American
MMI	Medical Mistrust Inventory
GBMMS	Group-Based Medical Mistrust Scale
SES	Socioeconomic status
GED	Graduate equivalency diploma
HS	High school
SILS	Single-item literacy screener
HINTS	Health Information National Trends Survey
SD	Standard deviation
IQR	Interquartile range
