# Supplementary material for: Prevalence and Predictors of Medical Mistrust Among Socioeconomically and Racially Diverse Cancer Patients in Philadelphia
Source: Cancers (Basel). 2025 Feb 14;17(4):649. doi: 10.3390/cancers17040649 (PMC11853404; doi:10.3390/cancers17040649)
Supplement: Supplementary file 1 [file cancers-17-00649-s001.zip › cancers-3428595-supplementary.pdf]

Supplementary tables and figure

Supplementary Table S1. Association of demographic factors with race-based mistrust (GBMMS) subscales of suspicion, discrimination, and lack of support.

	GBMMS Suspicion			GBMMS Discrimination			GBMMS Lack of support		
	**Median**	**Mean (SD)**	**p**	**Median**	**Mean (SD)**	**p**	**Median**	**Mean (SD)**	**p**
**Treatment site**			0.021			0.82			0.035
Urban campus	1.83	1.99 (0.86)		2.67	2.59 (1.14)		2.33	2.32 (0.95)	
Suburban campus	1.17	1.69 (0.94)		2.33	2.55 (1.15)		2.00	2.04 (0.87)	
**Age (years)**			0.095			0.13			0.088
25-49	1.82	1.95 (0.9)		3.00	2.88 (1.11)		2.33	2.31 (0.89)	
50-59	2.00	2.04 (1.05)		2.33	2.41 (1.12)		2.33	2.37 (1.01)	
60-69	1.67	1.81 (0.96)		2.50	2.50 (1.21)		2.17	2.14 (0.93)	
70-91	1.33	1.58 (0.71)		2.00	2.37 (1.06)		1.67	1.92 (0.82)	
Missing	2.33	2.33		4.00	4.00		2.67	2.67	
**Race/ethnicity**			<0.001			<0.001			<0.001
Black/AA NH	2.00	2.14 (0.92)		3.00	2.9 (1.04)		2.67	2.59 (0.94)	
White NH	1.00	1.35 (0.69)		2.00	2.2 (1.1)		1.33	1.65 (0.66)	
Any Hispanic	1.83	1.83 (0.91)		2.00	2.44 (1.25)		2.00	2.13 (0.84)	
Other	2.67	2.38 (0.89)		3.00	2.58 (1.29)		2.33	2.25 (0.58)	
**Gender**			0.6			0.16			0.75
Female	1.67								
Male	1.50								
**Marital status**			0.76			0.22			0.039
Married	1.33			2.33			1.67		
Widowed	2.00			3.50			2.50		
Divorced/sep	1.50			2.83			2.00		
Single	1.83			2.67			2.33		
Missing	2.33	2.33		2.00	2.00		2.00	2.00	
**Education**			0.79			0.30			0.066
< 9th grade	1.75			1.67			1.50		
Some High school	1.92			2.83			2.33		
HS diploma/GED	1.83			2.33			2.33		
Vocational	2.00			3.00			2.33		
Some college	1.33			2.67			2.33		
College grad	1.50			2.00			1.67		
Graduate degree	1.17			3.00			1.67		
**Income**			0.17			0.77			0.002
<$10,000	1.67	1.84 (0.86)		2.67	2.71 (1.15)		2.00	2.26 (0.9)	
$10K-<=25K	1.92	1.98 (0.95)		2.33	2.43 (1.19)		2.33	2.19 (0.79)	
$25K-<=50K	1.33	1.7 (0.75)		2.67	2.55 (1.15)		2.33	2.32 (0.81)	
$50K-<=75K	1.17	1.63 (0.94)		2.33	2.25 (0.89)		1.67	1.89 (0.9)	
$75K-<=100K	1.00	1.38 (0.6)		2.17	2.4 (1.07)		1.33	1.69 (0.79)	

$100K or more	1.08	1.71 (1.09)		2.83	2.72 (1.29)		1.33	1.72 (0.8)	
Don't know	2.17	2.29 (0.79)		3.00	2.83 (0.93)		2.67	2.94 (0.86)	
Decline	1.75	2.01 (0.99)		2.33	2.62 (1.25)		2.33	2.38 (1.09)	
**Insurance**			0.41			0.83			0.50
Private	1.17	1.71 (0.94)		2.50	2.52 (1.21)		2.00	2.09 (0.94)	
Medicare	1.67	1.72 (0.79)		2.67	2.68 (1.18)		2.33	2.09 (0.78)	
Medicaid	1.83	1.99 (0.96)		2.50	2.56 (1.07)		2.33	2.30 (1.00)	
Other	2.00	2.06 (0.85)		2.33	2.43 (1.01)		2.33	2.39 (1.02)	
More than one	1.33	1.8 (1.05)		2.00	2.41 (1.16)		1.67	2.03 (0.94)	
**Cancer stage**			0.030			0.14			0.063
Early stage	1.67	1.9 (0.98)		2.50	2.69 (1.27)		2.00	2.04 (0.99)	
Late stage	1.33	1.62 (0.76)		2.33	2.36 (1.03)		2.33	2.09 (0.83)	
Unsure	1.83	2.02 (0.98)		2.67	2.69 (1.13)		2.33	2.39 (0.92)	

Abbreviations: MMI, medical mistrust inventory; GBMMS, group-based medical mistrust scale; NH, non-Hispanic; HS, high school; GED, Graduate equivalency diploma; K, thousands.

Supplementary Table S2. Association of health literacy and trust in information sources with race-based discrimination (GBMMS) subscales suspicion, discrimination, and lack of support.

	**Suspicion**		**Discrimination**		**Lack of support**	
	**Mean**	**p**	**Mean**	**p**	**Mean**	**p**
**Health literacy**		0.22		0.59		0.4
Never need help with medication	1.85		2.54		2.20	
Any help with medication	1.79		2.64		2.08	
**Trust in information sources**						
**Doctor/health professional**		<0.001		<0.001		<0.001
Not at all/a little	2.45		3.1		2.82	
Some	2.31		3.09		2.76	
A lot	1.64		2.35		1.94	
**Radio**		0.15		0.032		0.036
Not at all	1.95		2.74		2.35	
A little	1.68		2.29		1.97	
Some/A lot	1.94		2.68		2.24	
**Government/health agencies**		0.17		0.01		0.014
Not at all	2.13		3.26		2.45	
A little	1.99		2.71		2.39	
Some	1.71		2.4		2.09	
A lot	1.76		2.44		2.06	
**Health organizations**		0.22		0.010		0.10
Not at all/ a little	2.08		3.06		2.48	
Some	1.77		2.57		2.11	
A lot	1.8		2.36		2.11	
**Patient testimonials**		0.44		0.03		0.26

Not at all	1.97	2.95	2.43
A little	1.66	2.70	2.31
Some	1.84	2.62	2.11
A lot	1.93	2.17	2.04
**Other sources**			
Family/Friends	0.36	0.51	0.24
Other cancer patients	0.21	0.66	0.21
Newspapers	0.49	0.46	0.73
Magazines	0.77	0.85	0.92
Internet	0.87	0.70	0.58
Television	0.09	0.70	0.99
Religious organizations	0.28	0.99	0.63
Charitable organizations	0.22	0.75	0.53

Abbreviations: GBMMS, group-based medical mistrust scale.

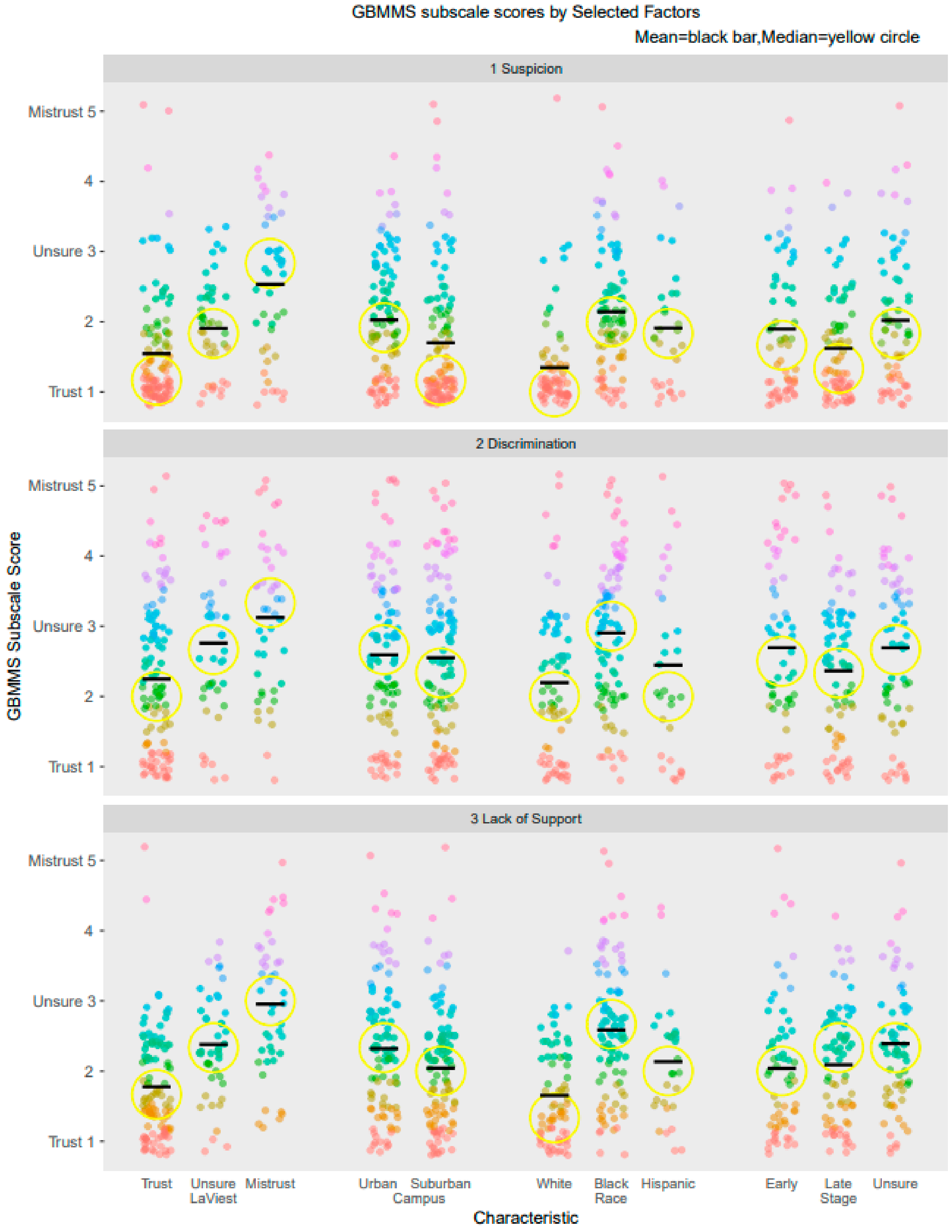

Figure S1. Distribution of GBMMS medical mistrust scores for LaVeist MMI, site of treatment, race and stage awareness stratified by suspicion, discrimination, and lack of support subscales.
